# Crystal structure of an unknown solvate of {2,2′-[ethane-1,2-diylbis(nitrilo­methanylyl­idene)]diphenolato-κ^4^ *O*,*N*,*N*′,*O*′}(*N*-ferrocenylisonicotinamide-κ*N* ^1^)cobalt(II): a Co^II^–salen complex that forms hydrogen-bonded dimers

**DOI:** 10.1107/S2056989015014723

**Published:** 2015-08-26

**Authors:** Bryan Brautigam, Chelsea Herholdt, William Farnsworth, Ellen Brudi, Eric McDonald, Guang Wu, Stephen Contakes

**Affiliations:** a Department of Chemistry, Westmont College, 955 La Paz Road, Santa Barbara, CA 93108, USA; b UCSB College of Letters and Science, X-Ray Analytical Facility, 4610 Physical Sciences North, UC Santa Barbara, Santa Barbara CA 93106, USA

**Keywords:** crystal structure, salen complex, pyridine ligand, ferrocene, bimetallic, hydrogen-bonded dimer

## Abstract

The title compound, [CoFe(C_5_H_5_)(C_16_H_14_N_2_O_2_)(C_11_H_9_N_2_O)], was prepared as an air-stable red–brown solid by mixing equimolar amounts of {2,2′-[ethane-1,2-diylbis(nitrilo­methanylyl­idene)]diphenolato}cobalt(II) and *N*-ferrocenylisonicotinamide in dry di­chloro­methane under nitro­gen and was characterized by ESI–MS, IR, and single-crystal X-ray diffraction. The structure at 100 K has triclinic (*P*-1) symmetry and indicates that the complex crystallizes as a mixture of λ and δ conformers. It exhibits the expected square pyramidal geometry about Co, and forms hydrogen-bonded dimers through amide N—H groups and phenolate O atoms on an adjacent mol­ecule. The involvement of only half of the salen ring structure in hydrogen-bonding inter­actions results in slight folding of the salen ring away from the pyridine coordination site in the δ conformer with an inter-salicyl­idene fold angle of 9.9 (7)°. In contrast, the λ conformer is nearly planar. The dimers pack into an open structure containing channels filled with highly disordered solvent mol­ecules. These solvent molecules’ contributions to the intensity data were removed with the SQUEEZE procedure [Spek (2015). *Acta Cryst.* C**71**, 9–18] available in *PLATON*.

## Abstract 2

The title cobalt(II) complex was prepared by mixing equimolar amounts of {2,2′-[ethane-1,2-diylbis(nitrilo­methanylyl­idene)]diphenolato}cobalt(II) and *N*-ferrocenylisonicotinamide in dry di­chloro­methane under nitro­gen and subsequently characterized by ESI–MS, IR, and single-crystal X-ray diffraction. The structure at 100 K has triclinic (*P* 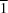) symmetry and indicates that the complex crystallizes as a mixture of λ and δ conformers. It exhibits the expected square-pyramidal geometry about the Co^II^ atom, and forms hydrogen-bonded dimers through the amide N—H group and one of the phenolate O atoms on adjacent mol­ecules.

## Chemical context   

Ferrocenes have been studied extensively on account of their stable sandwich structure, ability to undergo reversible one-electron oxidation, and, more recently, their potential utility in asymmetric catalysis applications (Stepnicka, 2008 ▸; Dai & Hou, 2010 ▸). *N*-Ferrocenyl­amides in particular have been investigated for their ability to form hydrogen bonds through the amide N—H group and carbonyl O atom. They have been employed to construct hydrogen-bonding scaffolds (Okamura *et al.*, 1998 ▸; Barisić *et al.*, 2006 ▸), perturb the redox properties of attached metal atoms (Okamura *et al.*, 2007 ▸), and form one-dimensional hydrogen-bonded chains that can support fast electron transfer (Okamura *et al.*, 2005 ▸). We recently demonstrated that the hydrogen-bond network in one of these systems, *N*-ferrocenylisonicotinamide, is able to support a mixed-valent state in the solid (Patterson *et al.*, 2015 ▸). Inter­estingly, the hydrogen bonds in *N*-ferrocenylisonicotinamide occur exclusively between the amide N—H group and the amide carbonyl O atom; the pyridyl N atom of the isonicotinoyl group is not involved. This suggested that it might be possible to use the pyridine N atom to coordinate metal atoms while leaving the isonicotinamide amide group free to form hydrogen-bonded chains or otherwise engage in hydrogen-bonding inter­actions. To this end, a complex between *N*-ferrocenylisonicotinamide and {2,2′-[ethane-1,2-diylbis(nitrilo­methanylyl­idene)]diphenolato}cobalt(II) was prepared and its structure determined.
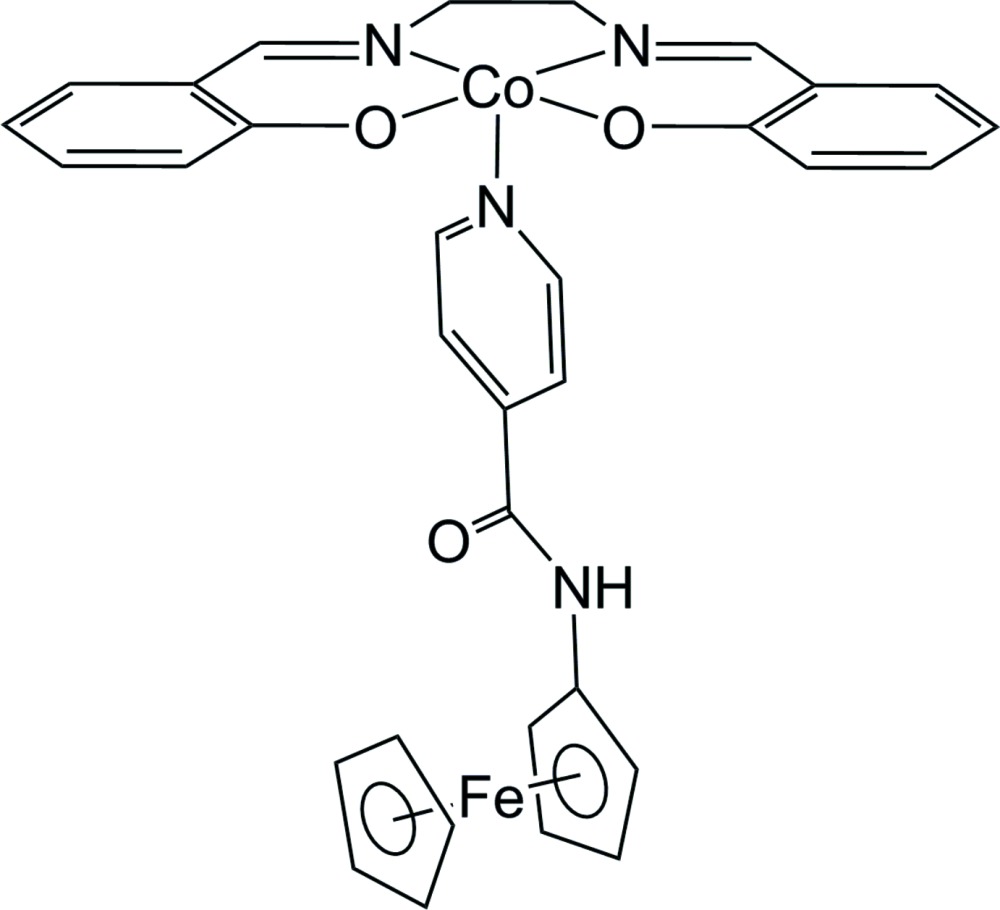


A cobalt complex of *N*,*N*′-bis­(salicyl­idene)ethyl­enedi­amine (salen) was selected for this study since Co^II^(salen), and its derivatives are known to function as oxygen carriers (Tsumaki, 1938 ▸; Calvin *et al.*, 1946 ▸; Chen *et al.*, 1989 ▸), both as solids and in solution. Complexes with pyridine-based ligands are of particular inter­est since the ability of Co^II^(salen) to absorb oxygen in di­chloro­methane or chloro­form solutions is dependent on the presence of pyridine or other co-ligands to complete the *d* ^6^ Co^III^(salen)-superoxide complex’s octa­hedral coordination sphere. The system also permits structural comparisons with reported crystal structures of oxygen-active (Schaefer and Marsh, 1969 ▸) and inactive (Holt *et al.*, 1971 ▸) forms of Co^II^(salen) and with the products of its reaction with oxygen, κ^1^-superoxido-complexes (Floriani & Calderazzo, 1969 ▸; Schaefer *et al.*, 1980 ▸) and peroxido-bridged dimers (Floriani & Calderazzo, 1969 ▸; Fritch *et al.*, 1973 ▸). Chiral and achiral salens and their derivatives are also of inter­est due to their ability to function as versatile catalysts for a variety of oxidation, ring opening, hydrolysis, and polymerization applications (Zhang *et al.*, 1990 ▸; Yoon & Jacobsen, 2003 ▸; Cozzi, 2004 ▸; Darensbourg, 2007 ▸; Gupta & Sutar, 2008 ▸; Ou & Wu, 2014 ▸). Cobalt–salen complexes in particular are used to catalyze the ring opening and hydrolysis of epoxides (Tokunaga *et al.*, 1997 ▸; Schaus *et al.*, 2002 ▸; Ford *et al.*, 2013 ▸; Crossley *et al.*, 2014 ▸; White *et al.*, 2014 ▸) and the oxidation of phenols (Van Dort & Geursen, 1967 ▸).

## Structural commentary   

**Figure 1:**
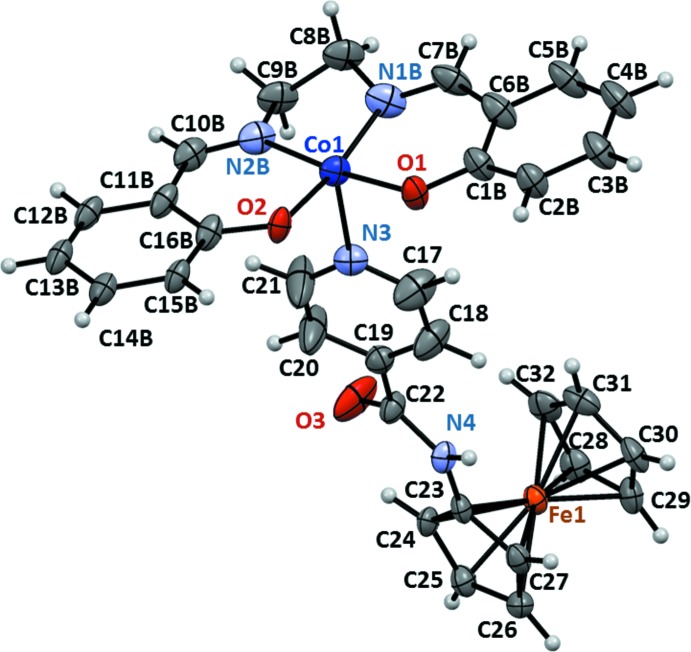
The asymmetric unit of the title compound, showing the atom-naming scheme. Only the δ conformer is depicted for clarity. The displacement ellipsoids are shown at the 50% probability level.

The title compound crystallizes as a 58.3 (12)/41.7 (12)% mixture of its λ and δ chelate ring conformers. In both cases the coordination environment of the Co^II^ ion is roughly square pyramidal (Fig. 1 ▸), although the Co^II^ ion is displaced 0.15 Å away from the N_2_O_2_ plane of the salen and towards the axial N atom. A similar 0.20 Å displacement is observed in the structure of [Co^II^(salen)(py)] (Calligaris *et al.*, 1970 ▸). The average Co^II^–N_eq_ and Co^II^—O bond lengths in the present complex are 1.88 and 1.90 Å, both significantly shorter than the Co—N_ax_ bond length of 2.159 (4) Å. The equatorial bond lengths are in good agreement with those observed for other pyridine complexes of Co(salen), although the axial Co—N bond length is more similar to the 2.10 (2) Å distance observed for [Co^II^(salen)(py)] than the shorter 1.896 Å distance observed for the more highly oxidized [Co^III^(salen)(py)_2_]^+^ (Shi *et al.*, 1995 ▸).

The axial py group in the present complex exhibits considerable librational mobility associated with its ability to rotate about the Co—N and C-amide bonds. In fact, the average twist angle between the pyridine and amide planes is 28.2 (2)°, suggesting that the two are not tightly coupled electronically; in contrast, the amide and Cp are tightly coupled with a N—C(Cp) distance of 1.395 (5) Å and an inter­plane twist angle of 4.83 (3)°. Similar behavior is observed in the structure of *N*-ferrocenylisonicotinamide itself (Patterson *et al.*, 2015 ▸).

## Supra­molecular Features   

**Figure 2:**
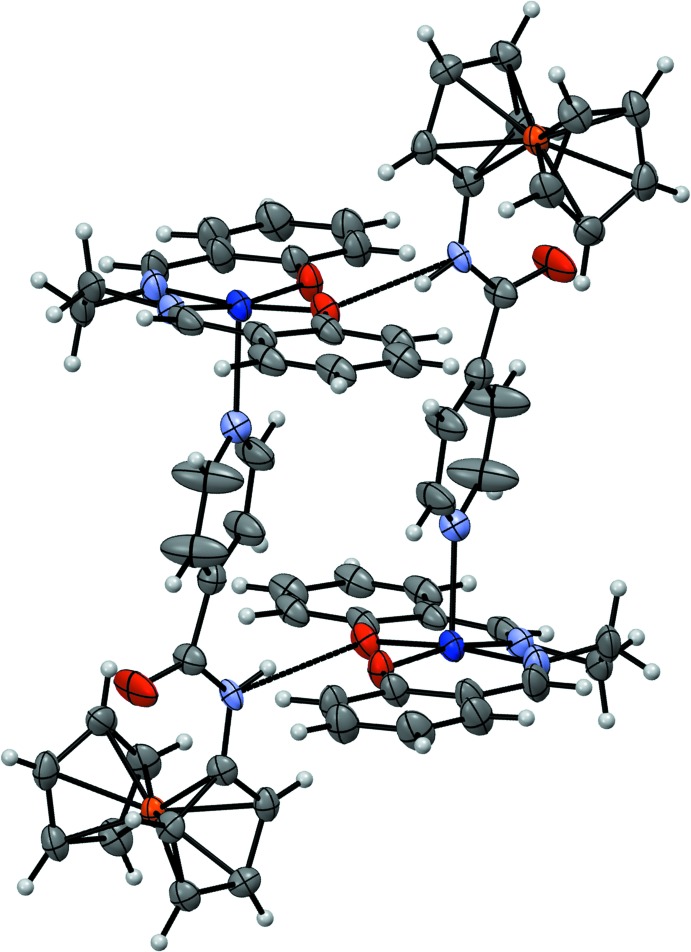
Hydrogen-bonded dimers of the title compound, showing the hydrogen bonds (dashed lines) formed between amide N—H groups and phenolate O atoms. Only the λ conformers are depicted for clarity. The displacement ellipsoids are shown at the 50% probability level.

Mol­ecules of the title compound form dimers in the solid state (Fig. 2 ▸). These are linked by hydrogen bonds between the amide N—H group and one of the phenolate O atoms on adjacent mol­ecules. Inter­action between these atoms is facilitated by twisting of the *N*-ferrocenylisonicotinamide amide group so that the amide plane (and presumably the amide N—H group) is oriented towards one of the two phenolate O atoms on the adjacent complex. The N⋯O distance for this inter­action is 2.799 (4) Å, within the typical range for medium strength hydrogen bonds (Steiner, 2002 ▸) and shorter than the 2.969 (4) Å distance between the amide N and the other phenolate O atom. The Co—O distance to the hydrogen-bonded O atom, 1.908 (3) Å, is slightly longer than the 1.885 (3) Å distance between Co^II^ and the other phenolate O atom. The amide N—H⋯O hydrogen-bond angle is 151.3°, smaller than the 164.0 (2)° angle observed in the structure of the *N*-ferrocenylisonicotinamide ligand (Patterson *et al.*, 2015 ▸) and within the range of those observed for aliphatic *N*-ferrocenyl­amides engaged in N—H⋯O=C hydrogen bonding (Okamura *et al.*, 2005 ▸).

**Figure 3:**
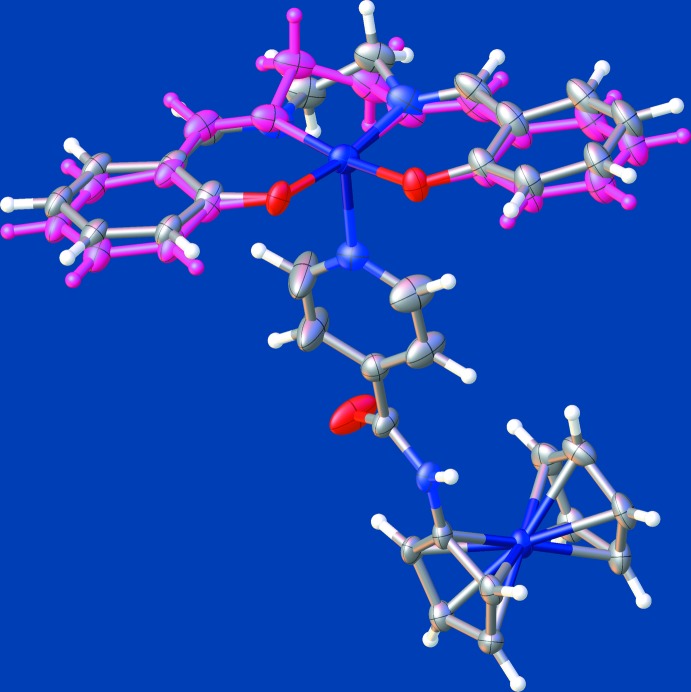
Comparison of the salen ring structure in the λ and δ conformers of the title compound, showing the greater bowing of salen in the latter. The salen ring system of the δ conformer is colored by element, while that of the λ conformer is shown in purple.

The involvement of only half of the salen ring structure in hydrogen-bonding inter­actions means that the λ and δ conformers are diastereomeric. In the δ conformer, the salen ring is slightly folded away from the py coordination site (Fig. 3 ▸), with an inter­salicyl­idene fold angle of 9.9 (7)°. In contrast, the λ conformer is nearly planar with an inter­salicyl­idene fold angle of 2.3 (5)°. The discrepancy between the λ and δ fold angles is consistent with the known flexibility of salen complexes. The related complex [Co^II^(salen)(py)], for instance, exhibits bending of the salen ring system away from the axial pyridine (py) ring with a fold angle of 28.8° (Calligaris *et al.*, 1970 ▸).

**Figure 4:**
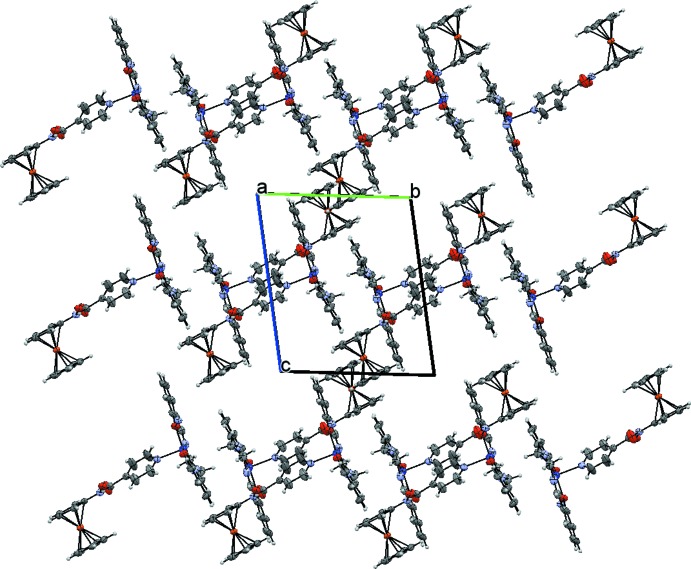
Packing structure view along the (100) axis of the crystal, showing the channels formed by packing of dimers of the title compound. Only the δ conformers are depicted for clarity.

The dimers pack into a layered structure along the [100] direction and perpendicular to the Co—py bond. In the crystal, the open coordination site of each Co(salen)py subunit is blocked by the imine C—H group of an adjoining dimer, an observation consistent with the solid state complex’s stability towards oxygen (in contrast dilute solutions of the complex react rapidly with oxygen). The structure contains large channels oriented along the [100] direction (Fig. 4 ▸). These channels are filled with highly-disordered solvent which we were unable to model. The *PLATON* (Spek, 2009 ▸) SQUEEZE (Spek, 2015 ▸) report indicated a solvent-accessible volume of 448.2 Å^3^ per cell, corresponding to 26.1% of the unit-cell volume, that is occupied by 144.9 electrons. However, SQUEEZE slightly underestimates the actual void volume since it assumes simultaneous occupancy of both conformers of the title compound. The void volumes calculated for the λ and δ conformers using a 1.2 Å probe radius are 432 and 505 Å^3^ per cell, corresponding to an occupancy-weighted average void volume of 462 Å^3^ per cell. These void volumes and electron counts are both much larger than would be expected from the 0.3 CH_2_Cl_2_ and 1.5 Et_2_O solvent mol­ecules per unit cell indicated by elemental analysis of the vacuum dried crystals. We suspect that 1.7 mol­ecules of di­chloro­methane solvent per unit cell are lost when the crystals are dried prior to elemental analysis. If the undried crystals contained 2 CH_2_Cl_2_ and 1.5 Et_2_O solvent mol­ecules per unit cell, a void volume and electron count of 470 Å^3^ per cell and 147 electrons per cell are expected, consistent with the expected void volume and SQUEEZE electron count results.

## Database survey   

For structural studies of *N*-ferrocenylisonicotinamide, see: Patterson *et al.* (2015 ▸). For structural studies on hydrogen-bonded assemblies of *N*-ferrocenyl­amides and the use of *N*-ferrocenyl­amides, see: Okamura *et al.* (1998 ▸, 2005 ▸); Patterson *et al.* (2015 ▸). For complexes involving *N*-ferrocenyl­amide derivatives of thiol­ate ligands, see: Okamura *et al.* (2007 ▸). For structural studies on {2,2′-[ethane-1,2-diylbis(nitrilo­methanylyl­idene)]diphenolato}cobalt(II) and its pyridine derivatives, see: Brückner *et al.* (1969 ▸); Calligaris *et al.* (1970 ▸, 1972 ▸); Shi *et al.* (1995 ▸). For a summary of the basic features of the stereochemistry of metal salen systems, see: Yamada (1999 ▸).

## Synthesis and crystallization   

All syntheses and purification steps were conducted under a nitro­gen atmosphere using degassed solvents. *N*-ferrocenylisonicotinamide was prepared as described previously (Patterson *et al.*, 2015 ▸). Aceto­nitrile, THF, and di­chloro­methane were purchased from VWR and purified using an HG Waters solvent purification system prior to use. All other reagents and solvents were obtained from VWR or Sigma–Aldrich in reagent grade or higher purity and used as received.

NMR spectra were obtained using either a Bruker Avance III 400MHz NMR spectrometer or a Bruker Avance 300MHz NMR spectrometer with gradient probe. All spectra were referenced relative to solvent peaks. Mass spectra were obtained on samples in HPLC grade MeOH using a Thermo-electron LCQ-Deca XP Mass Spectrometer. UV–Vis Spectra were obtained on anaerobic samples in quartz cuvettes using a Thermo Nicolet Evolution 300 UV–Vis spectrometer using the appropriate solvent as a blank. IR spectra were obtained using a Thermo Electron Nexus 470 FTIR.

For the preparation of the title compound, *N*-ferrocenyliso­nicotinamide (48.39 mg, 0.1580 mmol) and {2,2′-[ethane-1,2-diylbis(nitrilo­methanylyl­idene)]diphenolato}cobalt(II) (52.10 mg, 0.1602 mmol) were mixed with dry di­chloro­methane (5 ml) under nitro­gen. The resulting mixture was heated to 323 K for approximately 2 h, during which time the reactants dissolved to give a red solution. The reaction mixture was allowed to cool and then the solvent was removed using an oil-pump vacuum to give a waxy solid, which was washed with six 10 ml aliquots of dry diethyl ether, redissolved in 3 ml fresh di­chloro­methane, reprecipitated by the addition of diethyl ether (25 ml), and dried under vacuum for 18 h to give the product as a red–brown solid that is soluble in di­chloro­methane, aceto­nitrile, methanol, THF, acetone, DMF, and DMSO, but insoluble in water, ether, and hexa­nes. Yield: 60 mg (75.83%). ^1^H NMR (300 MHz, CDCl_3_): δ 2.427 (*br s*, 2H), 3.907 (*br s*), 4.013 (*br s*) (Note: the two preceding singlets were not fully resolved and integrated to a total of 6H), 4.714 (*br s*, 1H), 6.27 (*br s*, 1H), 6.966 (*br s*, 2H), 8.30 (*br s*, 2H), 14.422 (*br s*, 2H). ESI–MS (MeOH, positive ion): *m*/*z* 630.7, 325.1 and 307. Selected IR (KBr, cm^−1^): 3249, 3215 (NH), 1670 (amide C=O stretch). UV–Vis, concentrated in CH_2_Cl_2_: [λ_max_ (∊, *M* ^−1^ cm^−1^)]: 336 (15109), 406 (17461), 482 (3674). Calculated for C_32_H_28_CoFeN_4_O_3_·0.75C_4_H_10_O·0.15 CH_2_Cl_2_: C 60.34, H 5.16, N 8.01%; found: C 60.39, H 5.21, N 8.06%.

Crystals were grown as opaque pale-red–brown plates by vapor diffusion of ethyl ether into a concentrated di­chloro­methane solution at 233 K under a nitro­gen atmosphere.

## Refinement   

Crystal data, data collection and structure refinement details are summarized in Table 1 ▸. H atoms were placed in idealized positions and refined as riding with bond lengths of 0.95 (CH), 0.98 (CH_2_), and 0.88 Å (amide NH). *U*_iso_(H) values were fixed at 1.2*U*_eq_(C). Several restraints were used to model the disorder associated with cocrystallization of the λ and δ chelate ring conformers. All phenolate C—O bond lengths were set to be equal using the SADI command and the phenolate C—O and its adjacent C atoms were constrained to be coplanar using the FLAT command. The displacement parameters of atoms C1*A* and C16*A* were set equal to those of C1*B* and C16*B* using the EADP command. The displacement parameter of all other disordered atoms (C2–C15) were constrained using the SIMU command.

After attempts to model the highly disordered solvent proved unsuccessful, the SQUEEZE technique (Spek, 2015 ▸) operated under *PLATON* (Spek, 2009 ▸) was used to filter out the contributions of the disordered solvent molecules, none of which was near the open coordination site on the Co^II^ atom, capable of forming hydrogen bonds with the N—H hydrogen or other O and N atoms in the structure, or otherwise within bonding distance to the mol­ecular structure.

Hydrogen-bond parameters were calculated assuming an ideal N—H bond angle and an N—H bond length of 1.009 Å, the value determined by neutron diffraction (Allen *et al.*, 2006 ▸). The void volumes expected for unit cells containing only the λ or δ chelate ring conformers were calculated in *Mercury3.3* (Macrae *et al.*, 2006 ▸) using the default probe radius of 1.2Å. The expected void volume occupancies for ether and di­chloro­methane mol­ecules were taken as 173 and 106 Å^3^, respectively, the average mol­ecular volume of each compound in the pure liquid at 293 K.

## Supplementary Material

Crystal structure: contains datablock(s) I. DOI: 10.1107/S2056989015014723/zl2632sup1.cif

Structure factors: contains datablock(s) I. DOI: 10.1107/S2056989015014723/zl2632Isup2.hkl

CCDC reference: 1417154

Additional supporting information:  crystallographic information; 3D view; checkCIF report

## Figures and Tables

**Table 1:** Experimental details

Crystal data	
Chemical formula	[CoFe(C_5_H_5_)(C_16_H_14_N_2_O_2_)(C_11_H_9_N_2_O)]
*M*_r_	631.36
Crystal system, space group	Triclinic, *P* 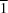
Temperature (K)	100
*a*, *b*, *c* ()	10.684(2), 11.989(3), 13.858(3)
, , ()	80.220(5), 85.234(5), 80.047(5)
*V* (^3^)	1720.2(7)
*Z*	2
Radiation type	Mo *K*
(mm^1^)	0.94
Crystal size (mm)	0.2 0.2 0.1

Data collection	
Diffractometer	Bruker SMART APEXII area detector
Absorption correction	Multi-scan (*SADABS*; Sheldrick, 2012 ▸)
*T*_min_, *T*_max_	0.639, 0.745
No. of measured, independent and observed [*I* > 2(*I*)] reflections	10667, 7295, 3968
*R*_int_	0.047
(sin /)_max_ (^1^)	0.644

Refinement	
*R*[*F* ^2^ > 2(*F* ^2^)], *wR*(*F* ^2^), *S*	0.056, 0.136, 0.93
No. of reflections	7295
No. of parameters	521
No. of restraints	638
H-atom treatment	H-atom parameters constrained
_max_, _min_ (e ^3^)	0.62, 0.58

Computer programs: *APEX2* (Bruker, 2005 ▸), *OLEX2.solve* (Bourhis *et al.*, 2015 ▸), *SHELXL2014* (Sheldrick, 2015 ▸) and *OLEX2* (Dolomanov *et al.*, 2009 ▸).

## supplementary crystallographic information

### Crystal data

**Table:**

[CoFe(C_5_H_5_)(C_16_H_14_N_2_O_2_)(C_11_H_9_N2O)]	*V* = 1720.2 (7) Å^3^
*M**_r_* = 631.36	*Z* = 2
Triclinic, *P*1	*F*(000) = 650
*a* = 10.684 (2) Å	*D*_x_ = 1.219 Mg m^−^^3^
*b* = 11.989 (3) Å	Mo *K*α radiation, λ = 0.71069 Å
*c* = 13.858 (3) Å	µ = 0.94 mm^−^^1^
α = 80.220 (5)°	*T* = 100 K
β = 85.234 (5)°	Plates, dull dark red
γ = 80.047 (5)°	0.2 × 0.2 × 0.1 mm

### Data collection

**Table:**

Bruker SMART APEXII area-detector diffractometer	7295 independent reflections
Radiation source: microfocus sealed X-ray tube, Incoatec Iµs	3968 reflections with *I* > 2σ(*I*)
Mirror optics monochromator	*R*_int_ = 0.047
Detector resolution: 7.9 pixels mm^-1^	θ_max_ = 27.2°, θ_min_ = 1.5°
ω and φ scans	*h* = −13→11
Absorption correction: multi-scan (*SADABS2012*; Sheldrick, 2012)	*k* = −15→12
*T*_min_ = 0.639, *T*_max_ = 0.745	*l* = −17→17
10667 measured reflections	

### Refinement

**Table:**

Refinement on *F*^2^	Primary atom site location: iterative
Least-squares matrix: full	Secondary atom site location: difference Fourier map
*R*[*F*^2^ > 2σ(*F*^2^)] = 0.056	Hydrogen site location: inferred from neighbouring sites
*wR*(*F*^2^) = 0.136	H-atom parameters constrained
*S* = 0.93	*w* = 1/[σ^2^(*F*_o_^2^) + (0.0599*P*)^2^] where *P* = (*F*_o_^2^ + 2*F*_c_^2^)/3
7295 reflections	(Δ/σ)_max_ < 0.001
521 parameters	Δρ_max_ = 0.62 e Å^−^^3^
638 restraints	Δρ_min_ = −0.58 e Å^−^^3^

### Special details

**Table:**

Geometry. All e.s.d.'s (except the e.s.d. in the dihedral angle between two l.s. planes) are estimated using the full covariance matrix. The cell e.s.d.'s are taken into account individually in the estimation of e.s.d.'s in distances, angles and torsion angles; correlations between e.s.d.'s in cell parameters are only used when they are defined by crystal symmetry. An approximate (isotropic) treatment of cell e.s.d.'s is used for estimating e.s.d.'s involving l.s. planes.

### Fractional atomic coordinates and isotropic or equivalent isotropic displacement parameters (Å^2^)

**Table:**

	*x*	*y*	*z*	*U*_iso_*/*U*_eq_	Occ. (<1)
Co1	0.08639 (5)	0.29271 (5)	0.45247 (4)	0.03107 (18)	
Fe1	0.24831 (5)	−0.44739 (5)	0.91168 (4)	0.02567 (17)	
O1	−0.0897 (2)	0.2936 (2)	0.4787 (2)	0.0326 (7)	
O2	0.0587 (2)	0.2644 (2)	0.3252 (2)	0.0312 (7)	
N4	0.1996 (3)	−0.2891 (3)	0.7102 (2)	0.0255 (8)	
H4	0.1182	−0.2654	0.7009	0.033*	
N3	0.1421 (4)	0.1144 (3)	0.5130 (3)	0.0346 (9)	
O3	0.3940 (3)	−0.2369 (3)	0.6831 (3)	0.0619 (12)	
C1A	−0.1586 (16)	0.3192 (14)	0.5562 (10)	0.0333 (12)	0.583 (12)
C2A	−0.2883 (16)	0.3127 (16)	0.5624 (13)	0.036 (2)	0.583 (12)
H2A	−0.3230	0.2914	0.5085	0.047*	0.583 (12)
C3A	−0.3684 (14)	0.3352 (11)	0.6418 (11)	0.044 (2)	0.583 (12)
H3A	−0.4560	0.3294	0.6421	0.057*	0.583 (12)
C4A	−0.3204 (12)	0.3668 (11)	0.7221 (10)	0.048 (3)	0.583 (12)
H4A	−0.3750	0.3847	0.7770	0.062*	0.583 (12)
C5A	−0.1939 (12)	0.3714 (11)	0.7203 (10)	0.046 (3)	0.583 (12)
H5A	−0.1607	0.3888	0.7765	0.060*	0.583 (12)
C6A	−0.1090 (13)	0.3515 (17)	0.6382 (12)	0.039 (2)	0.583 (12)
C7A	0.0225 (12)	0.3585 (14)	0.6398 (13)	0.044 (3)	0.583 (12)
H7A	0.0495	0.3784	0.6973	0.053*	0.583 (12)
N1A	0.1100 (12)	0.340 (2)	0.5693 (16)	0.043 (3)	0.583 (12)
C8A	0.2460 (9)	0.3394 (10)	0.5832 (7)	0.044 (2)	0.583 (12)
H8AA	0.2872	0.2613	0.6108	0.053*	0.583 (12)
H8AB	0.2546	0.3924	0.6290	0.053*	0.583 (12)
C9A	0.3079 (9)	0.3779 (10)	0.4829 (7)	0.043 (2)	0.583 (12)
H9AA	0.2891	0.4624	0.4650	0.052*	0.583 (12)
H9AB	0.4014	0.3540	0.4827	0.052*	0.583 (12)
N2A	0.2519 (17)	0.321 (2)	0.4131 (10)	0.038 (2)	0.583 (12)
C10A	0.3152 (18)	0.3082 (18)	0.3311 (9)	0.040 (2)	0.583 (12)
H10A	0.3973	0.3298	0.3220	0.048*	0.583 (12)
C11A	0.2709 (18)	0.264 (3)	0.2535 (13)	0.0325 (19)	0.583 (12)
C12A	0.3569 (16)	0.2361 (13)	0.1739 (11)	0.038 (3)	0.583 (12)
H12A	0.4439	0.2423	0.1769	0.046*	0.583 (12)
C13A	0.3206 (15)	0.2009 (11)	0.0939 (10)	0.039 (3)	0.583 (12)
H13A	0.3801	0.1850	0.0412	0.050*	0.583 (12)
C14A	0.1927 (16)	0.1888 (12)	0.0914 (11)	0.040 (3)	0.583 (12)
H14A	0.1648	0.1665	0.0352	0.052*	0.583 (12)
C15A	0.1061 (16)	0.2088 (15)	0.1694 (13)	0.033 (3)	0.583 (12)
H15A	0.0207	0.1977	0.1664	0.043*	0.583 (12)
C16A	0.1422 (16)	0.245 (2)	0.2527 (13)	0.0322 (16)	0.583 (12)
C1B	−0.160 (2)	0.329 (2)	0.5527 (13)	0.0333 (12)	0.417 (12)
C2B	−0.293 (2)	0.334 (2)	0.5589 (18)	0.037 (3)	0.417 (12)
H2B	−0.3323	0.3116	0.5079	0.048*	0.417 (12)
C3B	−0.3678 (19)	0.3715 (16)	0.6359 (16)	0.043 (3)	0.417 (12)
H3B	−0.4570	0.3722	0.6381	0.056*	0.417 (12)
C4B	−0.3151 (17)	0.4085 (15)	0.7113 (14)	0.046 (3)	0.417 (12)
H4B	−0.3676	0.4328	0.7651	0.060*	0.417 (12)
C5B	−0.1880 (17)	0.4094 (15)	0.7069 (14)	0.044 (3)	0.417 (12)
H5B	−0.1516	0.4369	0.7568	0.058*	0.417 (12)
C6B	−0.1098 (18)	0.370 (3)	0.6290 (17)	0.041 (3)	0.417 (12)
C7B	0.0179 (17)	0.3844 (19)	0.6309 (18)	0.042 (3)	0.417 (12)
H7B	0.0406	0.4186	0.6826	0.050*	0.417 (12)
N1B	0.1056 (16)	0.354 (3)	0.567 (2)	0.043 (3)	0.417 (12)
C8B	0.2280 (12)	0.3908 (14)	0.5762 (11)	0.042 (2)	0.417 (12)
H8BA	0.2520	0.3734	0.6453	0.050*	0.417 (12)
H8BB	0.2234	0.4741	0.5531	0.050*	0.417 (12)
C9B	0.3209 (11)	0.3236 (14)	0.5133 (11)	0.042 (2)	0.417 (12)
H9BA	0.3946	0.3637	0.4927	0.050*	0.417 (12)
H9BB	0.3523	0.2470	0.5504	0.050*	0.417 (12)
N2B	0.256 (2)	0.311 (3)	0.4260 (13)	0.039 (3)	0.417 (12)
C10B	0.323 (3)	0.298 (3)	0.3468 (13)	0.036 (3)	0.417 (12)
H10B	0.4096	0.3080	0.3443	0.044*	0.417 (12)
C11B	0.277 (3)	0.271 (4)	0.2613 (19)	0.033 (2)	0.417 (12)
C12B	0.362 (2)	0.2614 (19)	0.1778 (14)	0.033 (3)	0.417 (12)
H12B	0.4465	0.2746	0.1805	0.040*	0.417 (12)
C13B	0.326 (2)	0.2343 (16)	0.0944 (14)	0.035 (3)	0.417 (12)
H13B	0.3862	0.2256	0.0407	0.046*	0.417 (12)
C14B	0.200 (2)	0.2192 (17)	0.0869 (16)	0.033 (3)	0.417 (12)
H14B	0.1751	0.2004	0.0283	0.043*	0.417 (12)
C15B	0.112 (2)	0.232 (2)	0.1659 (17)	0.030 (3)	0.417 (12)
H15B	0.0262	0.2232	0.1599	0.040*	0.417 (12)
C16B	0.148 (2)	0.257 (4)	0.2543 (18)	0.0322 (16)	0.417 (12)
C17	0.0672 (5)	0.0607 (4)	0.5791 (4)	0.0554 (16)	
H17	−0.0165	0.0991	0.5918	0.072*	
C18	0.1031 (4)	−0.0478 (4)	0.6307 (4)	0.0505 (15)	
H18	0.0448	−0.0828	0.6765	0.066*	
C19	0.2235 (4)	−0.1038 (4)	0.6149 (3)	0.0305 (10)	
C20	0.2989 (5)	−0.0493 (5)	0.5444 (4)	0.074 (2)	
H20	0.3826	−0.0861	0.5294	0.096*	
C21	0.2560 (6)	0.0567 (5)	0.4953 (4)	0.078 (2)	
H21	0.3109	0.0910	0.4459	0.093*	
C22	0.2794 (4)	−0.2170 (4)	0.6734 (3)	0.0311 (11)	
C23	0.2335 (4)	−0.3992 (4)	0.7623 (3)	0.0258 (10)	
C24	0.3578 (4)	−0.4609 (4)	0.7841 (3)	0.0276 (10)	
H24	0.4360	−0.4331	0.7670	0.036*	
C25	0.3430 (4)	−0.5707 (4)	0.8356 (3)	0.0302 (10)	
H25	0.4101	−0.6298	0.8583	0.039*	
C26	0.2107 (4)	−0.5779 (4)	0.8478 (3)	0.0293 (10)	
H26	0.1739	−0.6420	0.8799	0.038*	
C27	0.1441 (4)	−0.4710 (4)	0.8029 (3)	0.0281 (10)	
H27	0.0542	−0.4511	0.8006	0.036*	
C28	0.3275 (4)	−0.4656 (4)	1.0441 (3)	0.0337 (11)	
H28	0.4000	−0.5191	1.0659	0.044*	
C29	0.1990 (4)	−0.4846 (4)	1.0571 (3)	0.0347 (11)	
H29	0.1703	−0.5529	1.0891	0.045*	
C30	0.1218 (4)	−0.3836 (4)	1.0140 (3)	0.0363 (11)	
H30	0.0317	−0.3720	1.0124	0.047*	
C31	0.2011 (4)	−0.3030 (4)	0.9739 (3)	0.0371 (11)	
H31	0.1738	−0.2280	0.9401	0.048*	
C32	0.3285 (4)	−0.3527 (4)	0.9927 (3)	0.0371 (11)	
H32	0.4015	−0.3169	0.9742	0.048*	

### Atomic displacement parameters (Å^2^)

**Table:**

	*U*^11^	*U*^22^	*U*^33^	*U*^12^	*U*^13^	*U*^23^
Co1	0.0191 (3)	0.0351 (4)	0.0402 (4)	−0.0031 (3)	−0.0074 (3)	−0.0078 (3)
Fe1	0.0189 (3)	0.0331 (4)	0.0253 (3)	−0.0017 (3)	−0.0004 (3)	−0.0086 (3)
O1	0.0198 (15)	0.0488 (19)	0.0320 (16)	−0.0056 (13)	−0.0019 (13)	−0.0138 (15)
O2	0.0155 (15)	0.0455 (19)	0.0311 (16)	−0.0072 (13)	0.0018 (12)	−0.0009 (14)
N4	0.0130 (17)	0.035 (2)	0.0276 (19)	−0.0022 (15)	−0.0042 (14)	−0.0027 (16)
N3	0.034 (2)	0.034 (2)	0.035 (2)	−0.0041 (18)	−0.0082 (18)	−0.0044 (18)
O3	0.0157 (18)	0.052 (2)	0.108 (3)	−0.0075 (15)	−0.0101 (19)	0.021 (2)
C1A	0.027 (2)	0.043 (3)	0.030 (2)	0.004 (2)	−0.0060 (18)	−0.014 (2)
C2A	0.028 (3)	0.048 (6)	0.035 (4)	0.001 (4)	−0.004 (3)	−0.020 (4)
C3A	0.031 (3)	0.058 (6)	0.044 (4)	0.001 (4)	−0.003 (3)	−0.021 (5)
C4A	0.039 (4)	0.061 (6)	0.042 (4)	0.008 (5)	−0.001 (3)	−0.021 (5)
C5A	0.039 (4)	0.057 (6)	0.042 (4)	0.010 (4)	−0.009 (3)	−0.026 (4)
C6A	0.030 (3)	0.050 (5)	0.038 (4)	0.007 (3)	−0.009 (3)	−0.021 (4)
C7A	0.039 (4)	0.052 (6)	0.047 (4)	0.008 (4)	−0.019 (3)	−0.028 (4)
N1A	0.028 (3)	0.048 (6)	0.057 (4)	0.003 (3)	−0.016 (3)	−0.024 (4)
C8A	0.028 (4)	0.048 (5)	0.064 (4)	0.003 (4)	−0.020 (3)	−0.028 (4)
C9A	0.026 (3)	0.035 (5)	0.072 (4)	−0.001 (3)	−0.019 (3)	−0.015 (4)
N2A	0.022 (3)	0.032 (4)	0.061 (4)	−0.001 (3)	−0.012 (3)	−0.012 (4)
C10A	0.019 (4)	0.036 (4)	0.059 (4)	−0.001 (3)	−0.005 (4)	0.007 (4)
C11A	0.019 (3)	0.033 (4)	0.040 (4)	−0.003 (3)	−0.002 (3)	0.009 (3)
C12A	0.020 (3)	0.040 (6)	0.046 (4)	−0.001 (4)	0.004 (3)	0.011 (4)
C13A	0.024 (3)	0.042 (6)	0.041 (3)	−0.001 (5)	0.007 (3)	0.010 (4)
C14A	0.031 (4)	0.045 (6)	0.037 (4)	−0.002 (5)	0.003 (3)	0.005 (4)
C15A	0.021 (3)	0.039 (6)	0.033 (3)	−0.002 (4)	0.001 (3)	0.006 (4)
C16A	0.019 (2)	0.037 (4)	0.035 (2)	−0.002 (2)	−0.0026 (18)	0.007 (2)
C1B	0.027 (2)	0.043 (3)	0.030 (2)	0.004 (2)	−0.0060 (18)	−0.014 (2)
C2B	0.027 (4)	0.051 (6)	0.035 (4)	0.000 (4)	−0.006 (4)	−0.018 (5)
C3B	0.034 (4)	0.054 (7)	0.041 (4)	0.009 (5)	−0.001 (4)	−0.021 (5)
C4B	0.041 (4)	0.055 (7)	0.042 (4)	0.011 (5)	−0.003 (4)	−0.027 (5)
C5B	0.041 (4)	0.053 (6)	0.040 (5)	0.013 (5)	−0.014 (4)	−0.025 (5)
C6B	0.033 (4)	0.051 (5)	0.040 (4)	0.010 (4)	−0.011 (3)	−0.021 (4)
C7B	0.038 (4)	0.044 (6)	0.047 (4)	0.005 (4)	−0.017 (4)	−0.025 (5)
N1B	0.028 (4)	0.047 (6)	0.056 (4)	0.002 (4)	−0.019 (4)	−0.017 (4)
C8B	0.029 (4)	0.042 (5)	0.060 (4)	−0.005 (4)	−0.019 (4)	−0.015 (5)
C9B	0.025 (4)	0.043 (5)	0.062 (5)	−0.005 (4)	−0.018 (4)	−0.012 (4)
N2B	0.022 (4)	0.036 (5)	0.058 (5)	0.001 (4)	−0.012 (4)	−0.006 (5)
C10B	0.017 (4)	0.035 (5)	0.054 (5)	−0.001 (4)	−0.008 (4)	0.001 (5)
C11B	0.017 (4)	0.034 (4)	0.042 (4)	−0.004 (3)	−0.002 (4)	0.008 (4)
C12B	0.017 (4)	0.034 (6)	0.044 (4)	−0.006 (4)	0.000 (4)	0.007 (4)
C13B	0.023 (4)	0.037 (6)	0.041 (4)	−0.007 (5)	0.005 (4)	0.007 (5)
C14B	0.028 (4)	0.034 (6)	0.035 (4)	−0.009 (5)	0.002 (4)	0.004 (5)
C15B	0.019 (4)	0.036 (6)	0.032 (4)	−0.007 (4)	0.001 (4)	0.005 (4)
C16B	0.019 (2)	0.037 (4)	0.035 (2)	−0.002 (2)	−0.0026 (18)	0.007 (2)
C17	0.024 (3)	0.046 (3)	0.089 (4)	0.000 (2)	−0.015 (3)	0.010 (3)
C18	0.021 (3)	0.045 (3)	0.078 (4)	−0.005 (2)	−0.003 (2)	0.012 (3)
C19	0.028 (2)	0.033 (3)	0.032 (2)	−0.007 (2)	−0.0021 (19)	−0.006 (2)
C20	0.053 (4)	0.046 (4)	0.097 (5)	0.014 (3)	0.044 (3)	0.017 (3)
C21	0.071 (4)	0.050 (4)	0.080 (4)	0.020 (3)	0.045 (3)	0.026 (3)
C22	0.021 (2)	0.033 (3)	0.037 (3)	−0.0040 (19)	0.0037 (19)	0.001 (2)
C23	0.019 (2)	0.032 (3)	0.025 (2)	−0.0010 (18)	0.0054 (17)	−0.0078 (19)
C24	0.016 (2)	0.033 (3)	0.034 (2)	−0.0064 (18)	0.0063 (18)	−0.008 (2)
C25	0.023 (2)	0.036 (3)	0.031 (2)	0.0001 (19)	0.0014 (19)	−0.008 (2)
C26	0.032 (3)	0.027 (2)	0.029 (2)	−0.0067 (19)	0.0036 (19)	−0.006 (2)
C27	0.023 (2)	0.039 (3)	0.025 (2)	−0.0036 (19)	−0.0009 (18)	−0.013 (2)
C28	0.028 (3)	0.047 (3)	0.027 (2)	−0.003 (2)	−0.0073 (19)	−0.010 (2)
C29	0.032 (3)	0.048 (3)	0.022 (2)	−0.003 (2)	0.0039 (19)	−0.006 (2)
C30	0.027 (3)	0.048 (3)	0.033 (3)	0.007 (2)	0.003 (2)	−0.019 (2)
C31	0.045 (3)	0.032 (3)	0.034 (3)	0.004 (2)	−0.012 (2)	−0.013 (2)
C32	0.037 (3)	0.048 (3)	0.032 (3)	−0.009 (2)	−0.007 (2)	−0.018 (2)

### Geometric parameters (Å, º)

**Table:**

Co1—O1	1.885 (3)	C1B—C6B	1.421 (12)
Co1—O2	1.908 (3)	C2B—H2B	0.9500
Co1—N3	2.159 (4)	C2B—C3B	1.369 (13)
Co1—N1A	1.854 (18)	C3B—H3B	0.9500
Co1—N2A	1.877 (17)	C3B—C4B	1.396 (13)
Co1—N1B	1.89 (3)	C4B—H4B	0.9500
Co1—N2B	1.87 (2)	C4B—C5B	1.356 (13)
Fe1—C23	2.064 (4)	C5B—H5B	0.9500
Fe1—C24	2.052 (4)	C5B—C6B	1.406 (13)
Fe1—C25	2.039 (4)	C6B—C7B	1.408 (12)
Fe1—C26	2.035 (4)	C7B—H7B	0.9500
Fe1—C27	2.031 (4)	C7B—N1B	1.289 (13)
Fe1—C28	2.046 (4)	N1B—C8B	1.473 (14)
Fe1—C29	2.034 (4)	C8B—H8BA	0.9900
Fe1—C30	2.029 (4)	C8B—H8BB	0.9900
Fe1—C31	2.032 (4)	C8B—C9B	1.487 (13)
Fe1—C32	2.052 (4)	C9B—H9BA	0.9900
O1—C1A	1.300 (9)	C9B—H9BB	0.9900
O1—C1B	1.306 (12)	C9B—N2B	1.482 (14)
O2—C16A	1.311 (9)	N2B—C10B	1.275 (13)
O2—C16B	1.312 (12)	C10B—H10B	0.9500
N4—H4	0.8800	C10B—C11B	1.429 (13)
N4—C22	1.330 (5)	C11B—C12B	1.419 (13)
N4—C23	1.396 (5)	C11B—C16B	1.429 (12)
N3—C17	1.323 (6)	C12B—H12B	0.9500
N3—C21	1.317 (6)	C12B—C13B	1.354 (13)
O3—C22	1.220 (5)	C13B—H13B	0.9500
C1A—C2A	1.396 (10)	C13B—C14B	1.401 (13)
C1A—C6A	1.429 (10)	C14B—H14B	0.9500
C2A—H2A	0.9500	C14B—C15B	1.395 (12)
C2A—C3A	1.372 (10)	C15B—H15B	0.9500
C3A—H3A	0.9500	C15B—C16B	1.407 (13)
C3A—C4A	1.397 (11)	C17—H17	0.9500
C4A—H4A	0.9500	C17—C18	1.381 (6)
C4A—C5A	1.360 (10)	C18—H18	0.9500
C5A—H5A	0.9500	C18—C19	1.363 (6)
C5A—C6A	1.421 (10)	C19—C20	1.366 (6)
C6A—C7A	1.423 (10)	C19—C22	1.510 (6)
C7A—H7A	0.9500	C20—H20	0.9500
C7A—N1A	1.314 (10)	C20—C21	1.358 (7)
N1A—C8A	1.480 (11)	C21—H21	0.9500
C8A—H8AA	0.9900	C23—C24	1.432 (5)
C8A—H8AB	0.9900	C23—C27	1.415 (6)
C8A—C9A	1.523 (11)	C24—H24	0.9500
C9A—H9AA	0.9900	C24—C25	1.414 (5)
C9A—H9AB	0.9900	C25—H25	0.9500
C9A—N2A	1.490 (12)	C25—C26	1.425 (6)
N2A—C10A	1.292 (10)	C26—H26	0.9500
C10A—H10A	0.9500	C26—C27	1.422 (5)
C10A—C11A	1.424 (11)	C27—H27	0.9500
C11A—C12A	1.422 (10)	C28—H28	0.9500
C11A—C16A	1.432 (10)	C28—C29	1.422 (6)
C12A—H12A	0.9500	C28—C32	1.420 (6)
C12A—C13A	1.358 (11)	C29—H29	0.9500
C13A—H13A	0.9500	C29—C30	1.412 (6)
C13A—C14A	1.402 (10)	C30—H30	0.9500
C14A—H14A	0.9500	C30—C31	1.408 (6)
C14A—C15A	1.389 (10)	C31—H31	0.9500
C15A—H15A	0.9500	C31—C32	1.414 (6)
C15A—C16A	1.404 (10)	C32—H32	0.9500
C1B—C2B	1.403 (12)		
			
O1—Co1—O2	85.79 (12)	C1B—C2B—H2B	118.9
O1—Co1—N3	95.43 (13)	C3B—C2B—C1B	122.1 (14)
O1—Co1—N1B	93.8 (4)	C3B—C2B—H2B	118.9
O2—Co1—N3	95.16 (13)	C2B—C3B—H3B	119.5
N1A—Co1—O1	94.4 (3)	C2B—C3B—C4B	121.1 (14)
N1A—Co1—O2	172.6 (9)	C4B—C3B—H3B	119.5
N1A—Co1—N3	92.2 (9)	C3B—C4B—H4B	120.4
N1A—Co1—N2A	86.5 (4)	C5B—C4B—C3B	119.2 (12)
N2A—Co1—O1	168.7 (8)	C5B—C4B—H4B	120.4
N2A—Co1—O2	91.8 (3)	C4B—C5B—H5B	119.9
N2A—Co1—N3	95.8 (8)	C4B—C5B—C6B	120.3 (13)
N1B—Co1—O2	167.8 (13)	C6B—C5B—H5B	119.9
N1B—Co1—N3	97.0 (13)	C5B—C6B—C1B	121.7 (11)
N2B—Co1—O1	173.1 (11)	C5B—C6B—C7B	113.4 (13)
N2B—Co1—O2	96.2 (4)	C7B—C6B—C1B	124.8 (13)
N2B—Co1—N3	91.0 (11)	C6B—C7B—H7B	118.2
N2B—Co1—N1B	82.9 (6)	N1B—C7B—C6B	123.7 (16)
C24—Fe1—C23	40.72 (15)	N1B—C7B—H7B	118.2
C24—Fe1—C32	108.71 (18)	C7B—N1B—Co1	127.4 (14)
C25—Fe1—C23	68.13 (16)	C7B—N1B—C8B	114.0 (17)
C25—Fe1—C24	40.46 (15)	C8B—N1B—Co1	117.7 (11)
C25—Fe1—C28	108.90 (17)	N1B—C8B—H8BA	110.8
C25—Fe1—C32	126.39 (18)	N1B—C8B—H8BB	110.8
C26—Fe1—C23	68.49 (16)	N1B—C8B—C9B	104.6 (12)
C26—Fe1—C24	68.76 (16)	H8BA—C8B—H8BB	108.9
C26—Fe1—C25	40.96 (16)	C9B—C8B—H8BA	110.8
C26—Fe1—C28	125.45 (17)	C9B—C8B—H8BB	110.8
C26—Fe1—C32	163.07 (17)	C8B—C9B—H9BA	110.0
C27—Fe1—C23	40.42 (16)	C8B—C9B—H9BB	110.0
C27—Fe1—C24	68.50 (16)	H9BA—C9B—H9BB	108.4
C27—Fe1—C25	68.50 (17)	N2B—C9B—C8B	108.5 (12)
C27—Fe1—C26	40.93 (16)	N2B—C9B—H9BA	110.0
C27—Fe1—C28	162.01 (17)	N2B—C9B—H9BB	110.0
C27—Fe1—C29	124.10 (18)	C9B—N2B—Co1	113.7 (12)
C27—Fe1—C31	119.63 (17)	C10B—N2B—Co1	126.7 (14)
C27—Fe1—C32	155.19 (17)	C10B—N2B—C9B	118.9 (16)
C28—Fe1—C23	156.71 (17)	N2B—C10B—H10B	117.6
C28—Fe1—C24	121.89 (17)	N2B—C10B—C11B	124.8 (16)
C28—Fe1—C32	40.55 (17)	C11B—C10B—H10B	117.6
C29—Fe1—C23	160.94 (16)	C12B—C11B—C10B	118.3 (14)
C29—Fe1—C24	156.65 (16)	C12B—C11B—C16B	118.3 (12)
C29—Fe1—C25	121.28 (17)	C16B—C11B—C10B	123.3 (14)
C29—Fe1—C26	106.84 (18)	C11B—C12B—H12B	119.0
C29—Fe1—C28	40.80 (16)	C13B—C12B—C11B	122.0 (13)
C29—Fe1—C32	68.46 (19)	C13B—C12B—H12B	119.0
C30—Fe1—C23	124.19 (17)	C12B—C13B—H13B	119.9
C30—Fe1—C24	161.74 (17)	C12B—C13B—C14B	120.2 (13)
C30—Fe1—C25	155.71 (18)	C14B—C13B—H13B	119.9
C30—Fe1—C26	119.56 (19)	C13B—C14B—H14B	120.1
C30—Fe1—C27	106.31 (18)	C15B—C14B—C13B	119.8 (13)
C30—Fe1—C28	68.32 (17)	C15B—C14B—H14B	120.1
C30—Fe1—C29	40.66 (16)	C14B—C15B—H15B	119.5
C30—Fe1—C31	40.59 (18)	C14B—C15B—C16B	121.0 (13)
C30—Fe1—C32	68.22 (19)	C16B—C15B—H15B	119.5
C31—Fe1—C23	107.38 (17)	O2—C16B—C11B	124.8 (19)
C31—Fe1—C24	125.44 (18)	O2—C16B—C15B	116.6 (18)
C31—Fe1—C25	162.81 (18)	C15B—C16B—C11B	118.6 (12)
C31—Fe1—C26	154.61 (18)	N3—C17—H17	117.9
C31—Fe1—C28	68.24 (18)	N3—C17—C18	124.1 (5)
C31—Fe1—C29	68.44 (18)	C18—C17—H17	117.9
C31—Fe1—C32	40.50 (17)	C17—C18—H18	120.5
C32—Fe1—C23	121.27 (18)	C19—C18—C17	119.0 (4)
C1A—O1—Co1	128.0 (9)	C19—C18—H18	120.5
C1B—O1—Co1	127.7 (12)	C18—C19—C20	116.5 (4)
C16A—O2—Co1	128.8 (9)	C18—C19—C22	124.5 (4)
C16B—O2—Co1	124.0 (12)	C20—C19—C22	119.0 (4)
C22—N4—H4	117.1	C19—C20—H20	119.5
C22—N4—C23	125.8 (3)	C21—C20—C19	121.1 (5)
C23—N4—H4	117.1	C21—C20—H20	119.5
C17—N3—Co1	120.8 (3)	N3—C21—C20	123.2 (5)
C21—N3—Co1	122.8 (3)	N3—C21—H21	118.4
C21—N3—C17	116.0 (4)	C20—C21—H21	118.4
O1—C1A—C2A	119.2 (13)	N4—C22—C19	117.3 (4)
O1—C1A—C6A	123.9 (13)	O3—C22—N4	124.3 (4)
C2A—C1A—C6A	116.9 (9)	O3—C22—C19	118.4 (4)
C1A—C2A—H2A	118.1	N4—C23—Fe1	128.1 (3)
C3A—C2A—C1A	123.7 (10)	N4—C23—C24	128.9 (4)
C3A—C2A—H2A	118.1	N4—C23—C27	123.4 (4)
C2A—C3A—H3A	120.2	C24—C23—Fe1	69.2 (2)
C2A—C3A—C4A	119.6 (10)	C27—C23—Fe1	68.5 (2)
C4A—C3A—H3A	120.2	C27—C23—C24	107.6 (4)
C3A—C4A—H4A	120.7	Fe1—C24—H24	126.0
C5A—C4A—C3A	118.7 (9)	C23—C24—Fe1	70.1 (2)
C5A—C4A—H4A	120.7	C23—C24—H24	126.2
C4A—C5A—H5A	118.4	C25—C24—Fe1	69.3 (2)
C4A—C5A—C6A	123.1 (10)	C25—C24—C23	107.7 (4)
C6A—C5A—H5A	118.4	C25—C24—H24	126.2
C5A—C6A—C1A	117.9 (8)	Fe1—C25—H25	126.3
C5A—C6A—C7A	120.8 (10)	C24—C25—Fe1	70.3 (2)
C7A—C6A—C1A	121.3 (10)	C24—C25—H25	125.6
C6A—C7A—H7A	116.9	C24—C25—C26	108.7 (3)
N1A—C7A—C6A	126.2 (12)	C26—C25—Fe1	69.4 (2)
N1A—C7A—H7A	116.9	C26—C25—H25	125.6
C7A—N1A—Co1	126.2 (10)	Fe1—C26—H26	126.1
C7A—N1A—C8A	121.2 (13)	C25—C26—Fe1	69.7 (2)
C8A—N1A—Co1	112.1 (8)	C25—C26—H26	126.4
N1A—C8A—H8AA	110.3	C27—C26—Fe1	69.4 (2)
N1A—C8A—H8AB	110.3	C27—C26—C25	107.1 (4)
N1A—C8A—C9A	107.2 (10)	C27—C26—H26	126.4
H8AA—C8A—H8AB	108.5	Fe1—C27—H27	125.3
C9A—C8A—H8AA	110.3	C23—C27—Fe1	71.1 (2)
C9A—C8A—H8AB	110.3	C23—C27—C26	108.8 (4)
C8A—C9A—H9AA	110.6	C23—C27—H27	125.6
C8A—C9A—H9AB	110.6	C26—C27—Fe1	69.7 (2)
H9AA—C9A—H9AB	108.8	C26—C27—H27	125.6
N2A—C9A—C8A	105.5 (9)	Fe1—C28—H28	126.4
N2A—C9A—H9AA	110.6	C29—C28—Fe1	69.1 (2)
N2A—C9A—H9AB	110.6	C29—C28—H28	126.0
C9A—N2A—Co1	113.5 (8)	C32—C28—Fe1	69.9 (2)
C10A—N2A—Co1	128.4 (10)	C32—C28—H28	126.0
C10A—N2A—C9A	117.7 (12)	C32—C28—C29	107.9 (4)
N2A—C10A—H10A	117.6	Fe1—C29—H29	125.9
N2A—C10A—C11A	124.8 (12)	C28—C29—Fe1	70.1 (2)
C11A—C10A—H10A	117.6	C28—C29—H29	126.2
C10A—C11A—C16A	122.4 (10)	C30—C29—Fe1	69.5 (2)
C12A—C11A—C10A	119.4 (10)	C30—C29—C28	107.7 (4)
C12A—C11A—C16A	118.2 (9)	C30—C29—H29	126.2
C11A—C12A—H12A	118.4	Fe1—C30—H30	126.1
C13A—C12A—C11A	123.1 (10)	C29—C30—Fe1	69.9 (2)
C13A—C12A—H12A	118.4	C29—C30—H30	125.8
C12A—C13A—H13A	121.0	C31—C30—Fe1	69.8 (2)
C12A—C13A—C14A	118.1 (10)	C31—C30—C29	108.4 (4)
C14A—C13A—H13A	121.0	C31—C30—H30	125.8
C13A—C14A—H14A	119.4	Fe1—C31—H31	125.7
C15A—C14A—C13A	121.2 (10)	C30—C31—Fe1	69.6 (3)
C15A—C14A—H14A	119.4	C30—C31—H31	125.8
C14A—C15A—H15A	119.3	C30—C31—C32	108.4 (4)
C14A—C15A—C16A	121.4 (10)	C32—C31—Fe1	70.5 (3)
C16A—C15A—H15A	119.3	C32—C31—H31	125.8
O2—C16A—C11A	121.8 (13)	Fe1—C32—H32	126.9
O2—C16A—C15A	120.3 (13)	C28—C32—Fe1	69.5 (2)
C15A—C16A—C11A	117.8 (9)	C28—C32—H32	126.2
O1—C1B—C2B	121.9 (17)	C31—C32—Fe1	69.0 (3)
O1—C1B—C6B	122.5 (18)	C31—C32—C28	107.6 (4)
C2B—C1B—C6B	115.5 (12)	C31—C32—H32	126.2
			
Co1—O1—C1A—C2A	−178.9 (8)	N2A—C10A—C11A—C16A	10 (4)
Co1—O1—C1A—C6A	−0.5 (16)	C10A—C11A—C12A—C13A	−175 (2)
Co1—O1—C1B—C2B	174.0 (12)	C10A—C11A—C16A—O2	−2 (4)
Co1—O1—C1B—C6B	−2 (2)	C10A—C11A—C16A—C15A	176 (3)
Co1—O2—C16A—C11A	−12 (3)	C11A—C12A—C13A—C14A	−2 (2)
Co1—O2—C16A—C15A	170.3 (12)	C12A—C11A—C16A—O2	177 (2)
Co1—O2—C16B—C11B	−1 (5)	C12A—C11A—C16A—C15A	−5 (4)
Co1—O2—C16B—C15B	177.9 (16)	C12A—C13A—C14A—C15A	−1.8 (16)
Co1—N3—C17—C18	−170.8 (4)	C13A—C14A—C15A—C16A	2 (2)
Co1—N3—C21—C20	169.5 (5)	C14A—C15A—C16A—O2	179.4 (18)
Co1—N1A—C8A—C9A	37.0 (19)	C14A—C15A—C16A—C11A	2 (3)
Co1—N2A—C10A—C11A	−3 (3)	C16A—C11A—C12A—C13A	5 (4)
Co1—N1B—C8B—C9B	−24 (3)	C1B—C2B—C3B—C4B	2 (3)
Co1—N2B—C10B—C11B	2 (5)	C1B—C6B—C7B—N1B	−5 (3)
Fe1—C23—C24—C25	59.3 (3)	C2B—C1B—C6B—C5B	2 (2)
Fe1—C23—C27—C26	−59.7 (3)	C2B—C1B—C6B—C7B	−172 (2)
Fe1—C24—C25—C26	58.9 (3)	C2B—C3B—C4B—C5B	1 (2)
Fe1—C25—C26—C27	59.5 (3)	C3B—C4B—C5B—C6B	−2 (2)
Fe1—C26—C27—C23	60.5 (3)	C4B—C5B—C6B—C1B	0 (3)
Fe1—C28—C29—C30	−59.5 (3)	C4B—C5B—C6B—C7B	175.4 (16)
Fe1—C28—C32—C31	58.6 (3)	C5B—C6B—C7B—N1B	180 (2)
Fe1—C29—C30—C31	−59.4 (3)	C6B—C1B—C2B—C3B	−3 (2)
Fe1—C30—C31—C32	−60.1 (3)	C6B—C7B—N1B—Co1	4 (4)
Fe1—C31—C32—C28	−58.9 (3)	C6B—C7B—N1B—C8B	173 (2)
O1—Co1—N1A—C7A	3 (2)	C7B—N1B—C8B—C9B	166 (2)
O1—Co1—N1A—C8A	174.1 (15)	N1B—Co1—O1—C1B	0.7 (16)
O1—Co1—N2A—C9A	87.6 (18)	N1B—Co1—N2B—C9B	18 (2)
O1—Co1—N2A—C10A	−85 (3)	N1B—Co1—N2B—C10B	−172 (3)
O1—Co1—N1B—C7B	−2 (3)	N1B—C8B—C9B—N2B	36 (3)
O1—Co1—N1B—C8B	−170 (2)	C8B—C9B—N2B—Co1	−36 (3)
O1—C1A—C2A—C3A	178.4 (15)	C8B—C9B—N2B—C10B	153 (2)
O1—C1A—C6A—C5A	−176.7 (15)	C9B—N2B—C10B—C11B	172 (4)
O1—C1A—C6A—C7A	0.4 (19)	N2B—Co1—N1B—C7B	172 (3)
O1—C1B—C2B—C3B	−180 (2)	N2B—Co1—N1B—C8B	4 (3)
O1—C1B—C6B—C5B	179 (2)	N2B—C10B—C11B—C12B	179 (3)
O1—C1B—C6B—C7B	4 (3)	N2B—C10B—C11B—C16B	2 (6)
O2—Co1—O1—C1A	−173.3 (10)	C10B—C11B—C12B—C13B	179 (3)
O2—Co1—O1—C1B	−167.0 (13)	C10B—C11B—C16B—O2	−2 (6)
O2—Co1—N2A—C9A	165.3 (14)	C10B—C11B—C16B—C15B	179 (4)
O2—Co1—N2A—C10A	−7.2 (19)	C11B—C12B—C13B—C14B	3 (3)
O2—Co1—N1B—C7B	86 (4)	C12B—C11B—C16B—O2	−179 (3)
O2—Co1—N1B—C8B	−83 (3)	C12B—C11B—C16B—C15B	2 (5)
O2—Co1—N2B—C9B	−174 (2)	C12B—C13B—C14B—C15B	0 (2)
O2—Co1—N2B—C10B	−4 (3)	C13B—C14B—C15B—C16B	−2 (3)
N4—C23—C24—Fe1	122.8 (4)	C14B—C15B—C16B—O2	−178 (2)
N4—C23—C24—C25	−177.9 (4)	C14B—C15B—C16B—C11B	1 (4)
N4—C23—C27—Fe1	−122.3 (4)	C16B—C11B—C12B—C13B	−3 (5)
N4—C23—C27—C26	178.0 (4)	C17—N3—C21—C20	−3.2 (10)
N3—Co1—O1—C1A	91.9 (10)	C17—C18—C19—C20	−3.0 (8)
N3—Co1—O1—C1B	98.2 (13)	C17—C18—C19—C22	173.5 (5)
N3—Co1—N1A—C7A	−93 (2)	C18—C19—C20—C21	2.0 (9)
N3—Co1—N1A—C8A	78.5 (16)	C18—C19—C22—N4	30.4 (7)
N3—Co1—N2A—C9A	−99.3 (14)	C18—C19—C22—O3	−150.3 (5)
N3—Co1—N2A—C10A	88.1 (19)	C19—C20—C21—N3	1.3 (11)
N3—Co1—N1B—C7B	−98 (3)	C20—C19—C22—N4	−153.2 (5)
N3—Co1—N1B—C8B	94 (3)	C20—C19—C22—O3	26.1 (7)
N3—Co1—N2B—C9B	−79 (2)	C21—N3—C17—C18	2.0 (8)
N3—Co1—N2B—C10B	91 (3)	C22—N4—C23—Fe1	90.0 (5)
N3—C17—C18—C19	1.1 (9)	C22—N4—C23—C24	−3.0 (7)
C1A—C2A—C3A—C4A	0.0 (18)	C22—N4—C23—C27	177.7 (4)
C1A—C6A—C7A—N1A	2 (2)	C22—C19—C20—C21	−174.7 (6)
C2A—C1A—C6A—C5A	1.6 (17)	C23—N4—C22—O3	−2.3 (7)
C2A—C1A—C6A—C7A	178.8 (13)	C23—N4—C22—C19	177.0 (4)
C2A—C3A—C4A—C5A	−1.7 (16)	C23—C24—C25—Fe1	−59.8 (3)
C3A—C4A—C5A—C6A	3.4 (16)	C23—C24—C25—C26	−0.9 (5)
C4A—C5A—C6A—C1A	−3.4 (18)	C24—C23—C27—Fe1	58.3 (3)
C4A—C5A—C6A—C7A	179.4 (12)	C24—C23—C27—C26	−1.4 (5)
C5A—C6A—C7A—N1A	178.8 (16)	C24—C25—C26—Fe1	−59.5 (3)
C6A—C1A—C2A—C3A	−0.1 (17)	C24—C25—C26—C27	0.1 (5)
C6A—C7A—N1A—Co1	−3 (3)	C25—C26—C27—Fe1	−59.7 (3)
C6A—C7A—N1A—C8A	−174.3 (16)	C25—C26—C27—C23	0.8 (5)
C7A—N1A—C8A—C9A	−151.1 (19)	C27—C23—C24—Fe1	−57.9 (3)
N1A—Co1—O1—C1A	−0.7 (12)	C27—C23—C24—C25	1.4 (5)
N1A—Co1—N2A—C9A	−7.5 (15)	C28—C29—C30—Fe1	59.9 (3)
N1A—Co1—N2A—C10A	180 (2)	C28—C29—C30—C31	0.5 (5)
N1A—C8A—C9A—N2A	−40.5 (17)	C29—C28—C32—Fe1	−58.9 (3)
C8A—C9A—N2A—Co1	28.8 (17)	C29—C28—C32—C31	−0.3 (5)
C8A—C9A—N2A—C10A	−157.9 (16)	C29—C30—C31—Fe1	59.5 (3)
C9A—N2A—C10A—C11A	−175 (2)	C29—C30—C31—C32	−0.6 (5)
N2A—Co1—O1—C1A	−95.1 (19)	C30—C31—C32—Fe1	59.5 (3)
N2A—Co1—N1A—C7A	171 (2)	C30—C31—C32—C28	0.6 (5)
N2A—Co1—N1A—C8A	−17.2 (16)	C32—C28—C29—Fe1	59.4 (3)
N2A—C10A—C11A—C12A	−170 (2)	C32—C28—C29—C30	−0.1 (5)
